# Phenotypic differentiation between wild and domesticated varieties of *Crescentia cujete* L. and culturally relevant uses of their fruits as bowls in the Yucatan Peninsula, Mexico

**DOI:** 10.1186/1746-4269-9-76

**Published:** 2013-11-14

**Authors:** Xitlali Aguirre-Dugua, Edgar Pérez-Negrón, Alejandro Casas

**Affiliations:** 1 Centro de Investigaciones en Ecosistemas, Universidad Nacional Autónoma de México, Campus Morelia, Apartado Postal 27-3 (Santa María de Guido), Morelia, Michoacán 58190, México

**Keywords:** Calabash, *Crescentia*, Domestication, Gourd tree, Maya, Mesoamerica, Morphology, Phenotypic variation

## Abstract

**Background:**

Selection criteria are important for analyzing domestication of perennial plant species, which experience a selection pressure throughout several human generations. We analyze the preferred morphological characteristics of *Crescentia cujete* fruits, which are used as bowls by the Maya of Yucatan, according to the uses they are given and the phenotypic consequences of artificial selection between one wild and three domesticated varieties.

**Methods:**

We performed 40 semi-structured interviews in seven communities. We calculated Sutrop’s salience index (*S*) of five classes of ceremonial and daily life uses, and of each item from the two most salient classes. We sampled 238 bowls at homes of people interviewed and compared their shape, volume and thickness with 139 fruits collected in homegardens and 179 from the wild. Morphology of varieties was assessed in fruit (*n* = 114 trees) and vegetative characters (*n* = 136 trees). Differences between varieties were evaluated through linear discriminant analysis (LDA).

**Results:**

Use of bowls as containers for the Day of the Dead offerings was the most salient class (*S* = 0.489) with chocolate as its most salient beverage (*S* = 0.491), followed by consumption of daily beverages (*S* = 0.423), especially maize-based *pozol* (*S* = 0.412). The sacred *saka’* and *balche'* are offered in different sized bowls during agricultural and domestic rituals. Roundness was the most relevant character for these uses, as bowls from households showed a strong selection towards round shapes compared with wild and homegarden fruits. Larger fruits from domesticated varieties were also preferred over small wild fruits, although in the household different sizes of the domesticated varieties are useful. LDA separated wild from domesticated trees (*p* < 0.001) according to both fruit and vegetative variables, but domesticated varieties were not different among themselves.

**Conclusions:**

The association between *C. cujete* bowls and traditional beverages in ritual and daily life situations has driven for centuries the selection of preferred fruit morphology in this tree. Selection of fruit roundness and volume has allowed for the differentiation between the wild variety and the three domesticated ones, counteracting gene flow among them. By choosing the best fruits from domesticated varieties propagated in homegardens, the Maya people model the domestication process of this important tree in their culture.

## Background

Domestication is an evolutionary process in which artificial selection increases fitness of individuals with morphological and physiological features favourable to people [1-3]. As the main evolutionary force driving domestication, artificial selection promotes phenotypic and genetic differentiation between domesticates and their wild relatives [4,5] with variable results according to intensity of past and current management practices of plant populations as well as features of the life history of managed plants. Plant management practices include gradients of activities with variable energy input, techniques and production goals in different geographical spaces. These practices include the tolerance or let standing, protection and promotion of preferred individuals in wild populations and their active cultivation in human-created environments such as agricultural parcels and homegardens [6]. In order to understand how artificial selection drives evolution of plant species under domestication, it is therefore necessary to analyze the environmental, social and cultural aspects influencing human management of biological variation [7].

Social and cultural aspects of plant management are particularly relevant to understand the decision-making processes that favour some individuals over others [6,8,9] because selection criteria and management practices depend on the role of each plant resource in a human culture. In the case of domestication of woody perennial plants, cultural criteria are linked to the process of intra-specific diversification of trees with cultivars for different purposes (e.g. in *Olea europaea* L. there exist approximately 1200 cultivars used for obtaining oil or olives, and in *Vitis vinifera* L. more than 5000 cultivars destined to wine, raisin or table grape production [10,11]). Markets and tourism are dynamic forces influencing the domestication process of trees, such as in the case of *Spondias tuberosa* Arruda in Brazil, in which fruit size is the main feature considered for collection intended for marketing purposes while flavour is prioritized when fruits are destined to domestic consumption [12]. Therefore, the variety of uses is undoubtedly linked to human selection that allows the phenotypic and genetic differentiation of perennial plant populations under domestication.

**Figure 1:**
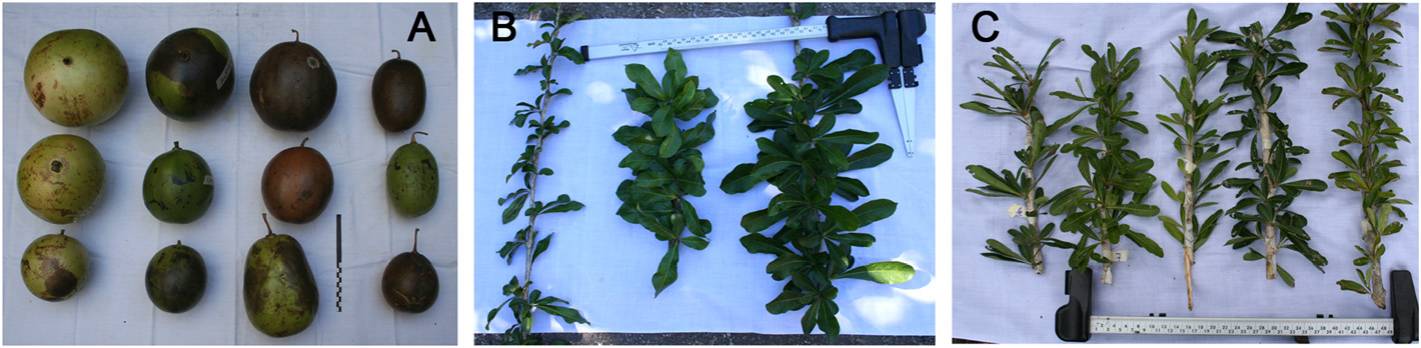
**Morphological variation in *Crescentia cujete *varieties from the study area. A)** Fruits (three first columns, from left to right) of *sac luch*, *yaax luch* and *uas* varieties growing in homegardens, and (fourth column) of *uas* variety growing in putative wild populations, scale: 20 cm; **B)** Leaves (from left to right) of *uas* wild variety and two individuals of *yaax luch* domesticated variety, all three growing in the same homegarden in Pachuitz; **C)** Leaves of *uas* wild variety growing in the putative wild population near Chun Ek; ruler in **B** and **C**: 50 cm.

*Crescentia cujete* L. (Bignoniaceae) is a tree species whose fruits were and currently are used by the Maya of the Yucatan Peninsula, Mexico, for preparing bowls commonly called *luch* in Maya and *jícaras* in Spanish. In that area, we previously studied the nomenclature of varieties recognized by local people, their frequency in wild and human-made environments, their management and genetic consequences of such management [13]. The Maya distinguish a wild variety called *uas* (güiro in Spanish) and three domesticated varieties, *luch* (jícara), *sac luch* (white jícara) and *yaax luch* (green jícara; Table 1), based on fruit size (domesticated varieties are larger than the wild one), roundness (domesticated fruits tend to be spherical while the wild variety produces elongated fruits) and pericarp thickness (domesticated varieties have a thicker pericarp; Figure 1). The wild variety is tolerated in some homegardens where it grows spontaneously while domesticated varieties are mainly clonally propagated in homegardens and a low proportion of trees are also cultivated through seed sowing (Table 1).

**Table 1:** ***Crescentia cujete *** **varieties found in the study area, their characteristics and management**

**Habitat**	**Variety**	**Main characteristics according to Maya people**	**Management**
Savanna	*uas*	Wild variety. Small, thin elongated fruits.	Spontaneous growth. Not harvested. Useful for shadow where the savanna is used as pastureland.
Homegarden	*uas*	Wild variety. Small, thin elongated fruits.	Spontaneous growth. Tolerated. Used occasionally for making bowls, mainly for shade or aesthetics, as well as a living fence and for its medicinal properties.
	*luch*	Domesticated variety. Round fruit, larger and thicker than wild variety.	Cultivated from cuttings, occasionally through seeds. Used mainly for making bowls, to a lesser extent for its medicinal properties.
	*sac luch*	Domesticated variety. Round fruit, light green when fresh, whitish aspect after being prepared, thinner pericarp.	Cultivated from cuttings. Used mainly for making bowls, sometimes for preparing remedies.
	*yaax luch*	Domesticated variety. “Not so round” fruit, dark green color when fresh, brownish aspect after preparation, thicker pericarp.	Cultivated from cuttings, occasionally through seeds. Used mainly for making bowls, but also for medicinal applications.

Table adapted from [13].

In contrast with most perennial plant species that have been domesticated for their edible fruits, *Crescentia cujete* is exclusively used for manufacturing bowls, being this and its sister species *C. alata* the only long-lived perennial plants whose fruits are used for manufacturing containers in the Americas [14,15]. Its fruit pulp is also commonly used in the Antilles and different regions of Mexico through Central and South America in the preparation of traditional remedies (usually in the form of a syrup) for treating respiratory ailments, internal abscesses and to expedite delivery [16-18]. In the Yucatan area, we observed that its leaves are used for fever relief (directly applied to the forehead) and the fruit pulp is prepared for treating respiratory illnesses.

The making of containers from *C. cujete* fruits represents a unique opportunity to study the cultural aspects motivating human selection of fruit morphological characters in association with the phenotypic differentiation between wild and cultivated varieties.

The communities of the Yucatan Peninsula represent the lowland area of the Maya people. These communities have long been recognized as exceptional reservoirs of biodiversity grounded on a diverse array of agricultural, horticultural and forestry strategies deeply rooted in the Maya culture and enriched with numerous plant species from the Old World [19-24]. Homegardens from the communities of the Yucatan Peninsula harbour nearly 350 plant species (mainly trees and shrubs), averaging 100–150 plant species per community [23,25], as well as domestic animals such as pigs, poultry (chickens, turkeys, ducks, doves) and native bees [19]. The biological richness, productivity and economic role of the Yucatan Maya homegardens [26-28] make them one of the spaces where social relationships are forged and reproduced (the other one being the *milpa*, which is the slash-and-burn agricultural plot where maize, beans and squash are grown together [29]).

Among the pre-Hispanic evidence of the role of *Crescentia cujete* fruits among the lowland Maya, it can be mentioned the presence of ornamented bowls in Chichén Itzá (1200–800 BP; [30]) and the representation of the species in ancient Maya vases [31]. Historical sources that point out their importance include the *Popol Vuh* (*ca.* 1544 AD), a mythical account about the creation of the world and epic tales. One of them refers that the head of one of the protagonists, Hun-Hunahpú, is placed by the lords of the underworld in a sterile tree that gets immediately covered by round fruits; then they “perceive the greatness of the essence of that tree [i.e. *C. cujete*]” [32]. The species was also described in the *Relación de las Cosas de Yucatán* by bishop Diego de Landa in 1566 AD: “There is a tree from whose fruit, which is like a round gourd, the Indians make their vessels, and they make them well painted and cute…”; also, “…from that [maize dough] they take a ball and dilute it in a glass made from the shell of a fruit that grows in a tree through which God provided them with glasses”. The Book of Chilam Balam of Chuyamel (ca. 1782), which includes religious and secular accounts, states the following sentence: “…‘My son, bring me your child, the one with the white-face, so I can see her, the one with the beautiful white head-dress’ (…) What he asks is the white *jícara* full of *saka’*  , water-of-maize-without-lime” [33]. Popenoe [34] includes *C. cujete* in a checklist of plant species from the region of Copán, Honduras, that were most probably as useful to the ancient Maya as they currently are to their descendants.

The primary goal of our study is to articulate the current uses of bowls made from *Crescentia cujete* fruits, the selected morphological characteristics of fruits associated with these uses, and the consequences of this selection on the phenotypic characteristics of trees. For this purpose, we integrated ethnobotanical and morphometric data from the trees growing in homegardens and in putatively wild populations. We aimed at: 1) documenting the diversity of uses of bowls made from *C. cujete* fruits in Maya communities and recognize how their characteristics influence human selection over the phenotypic variation of the species; 2) evaluating the morphological differentiation between varieties in fruit and vegetative characters; and 3) assessing how the observed phenotypic differentiation of these varieties is congruent with the species’ local nomenclature and the use of its fruits.

We expected that i) bowls used in households would reflect the characteristics preferred by people, mainly roundness, large size and a thick pericarp; ii) morphological differentiation between varieties will be more evident in fruit than in vegetative characters, as fruits are the direct targets of human selection; and iii) the use of bowls will be associated to fruit characteristics that define the Maya classification of the four varieties of the species.

## Methods

### Study area

**Figure 2:**
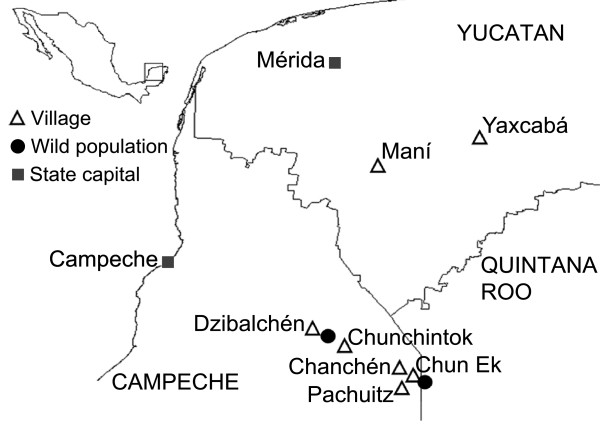
**Maya communities and *Crescentia cujete *** **wild populations of the study area in the Yucatan Peninsula, Mexico.**

Our study was conducted in seven Maya communities located in the states of Campeche and Yucatan, Mexico, and two putative wild sympatric populations growing in open savannas associated to semi-evergreen tropical forest (Figure 2, Table 2).

**Table 2:** Characteristics of the surveyed villages

**Village**	**Inhabitants**	**# Households**	**% Maya speakers**	**# Homegardens (interviews)**	**Altitude masl**	**Coordinates**	
						**N**	**W**
Yaxcabá	3007	722	56	6 (6)	30	20° 32′ 53″	−88° 49′ 38″
Maní	4146	982	76	8 (10)	20	20° 23′ 11″	−89° 23′ 25″
Dzibalchén	2340	591	34	14 (7)	170	19° 27′ 36″	−89° 43′ 42″
Chunchintok	1086	256	78	1 (1)	140	19° 21′ 35″	−89° 34′ 60″
Chun Ek	158	22	78	7 (6)	100	19° 11′ 13″	−89° 11′ 29″
Chan-Chen	304	53	79	7 (6)	120	19° 12′ 32″	−89° 15′ 41″
Pachuitz	266	50	86	4 (4)	140	19° 08′ 52″	−89° 14′ 56″

Data from [35].

### Ethnobotanical survey

In total, we visited 47 homegardens and performed 40 semi-structured interviews [36]. From these, 22 interviews were responded by women (15 in Spanish, 7 in Maya with the help of our local guide as translator), 14 by men (10 in Spanish, 4 in Maya) and 4 by man and woman couples (in Spanish). In this study, Maya spelling follows Barrera-Vásquez [37]. Although *jícaras* are common items in the study area, *Crescentia cujete* trees are not present in all households’ gardens, especially in Yaxcabá and Maní which are the most populated and urbanized villages among those surveyed (Table 2). Several authors [19,25,38-40] report this tree species as an element of the flora of lowland Maya homegardens at low frequency while other studies do not include it [41]. Distribution of the species in Maya homegardens is therefore variable among villages and among homegardens within a village (in our sample, mean number of trees per home = 1.7, mode = 1 tree). For the study, participants were selected using the snowball sampling method and by direct approach to the owners of homegardens where the tree species was identified. Our names and affiliations as well as the purpose of the study were introduced to people in order to obtain prior informed consent for conducting the interviews and for measuring trees and household bowls.

During the interviews we registered the daily and ceremonial uses of bowls described by the informants. We identified five main classes of uses as recipients: consumption of daily life beverages, other daily life uses (including scooping water for home activities and maize for feeding domestic animals), ceremonial offering of food and beverages for the Day of the Dead (November 1^st^ and 2^nd^), ceremonial offering of *saka’* and *balche’* during agricultural and domestic rituals, and ceremonial uses during other festivities. In order to identify the salience of each class of use, we considered the classes mentioned in each interview and calculated the Sutrop index (*S*) for each one of them with the formula *S = F / (N mP)*, where *F* represents the frequency of the term (in this case, the class), *N* is the total number of respondents, and *mP* is the mean position in which the term is named [42]. Subsequently, we analyzed the items of the two most salient classes: consumption of daily life beverages and ceremonial offerings for the Day of the Dead; the Sutrop index (*S*) was then calculated for every food item in each of these subsets of data. Calculations were performed through FLAME v1.0 software [43]. The techniques for preparing bowls and practices involved in bowls’ commercialization were also documented.

**Figure 3:**
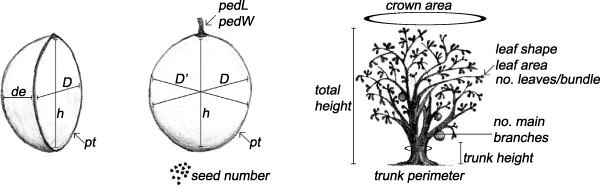
**Morphological measures taken on bowls, fruits and trees of *C. cujete*. *h*** **= height, *D*** **= major equatorial diameter, *D’*** **= minor equatorial diameter, *de*** **= depth, *pt*** **= pericarp thickness, *pedlL*** **= peduncle length, *pedW*** **= peduncle width.**

We requested access to the bowls present in interviewed people’s homes for taking the following measurements: height (*h*), diameter (*D*), depth (*de*) and bowl thickness (or pericarp thickness, *pt*; see Figure 3); *h*, *D* and *de* were measured in cm with a 50 cm scale with 1 mm precision, while *pt* was measured in mm with a 20 cm calliper with 0.02 mm precision. In order to estimate the shape of the bowl a roundness index was defined as *rd = (h/D)*, with values close to 1 in bowls with a round shape, values >1 if the shape corresponds to a vertical ellipse and values <1 if it corresponds to a horizontal ellipse. Bowl volume (in litres, L) was calculated with the formula of ellipsoid bodies *V =* [*4/3π (h/2)(D/2)(de)*/1000]/2. These measurements were made in a total of 258 bowls from 30 of the households visited.

Means and standard deviations, as well as the coefficient of variation (CV), were calculated for each variable. We also compared bowl characteristics (*rd, V* and *pt*) with fruits collected in homegarden trees and in the putative wild populations.

### Morphometric analyses

A total of 318 fruits were collected from 114 trees (179 fruits from 60 wild trees, 139 fruits from 54 trees growing in homegardens; mean number of fruits/tree = 2.8). We measured in the field the height of the fruit from the peduncle scar to the base (*h*), the major equatorial diameter (*D*) and the minor equatorial diameter (*D’*) in order to estimate the roundness of the fruit with the index *rd = (h/D)*. The volume of the fruit (*V*, in litres, L) was calculated with the formula of ellipsoid bodies volume *V = 4/3π(h/2)(D/2)(D’/2)*/1000. The thickness of the pericarp (*pt*) was measured in the major equatorial diameter and the length and width of the peduncle were also measured in mm using a 20 cm calliper with 0.02 mm precision (Figure 3). Seeds were extracted from one half of every fruit and directly counted. Mean fruit morphology was calculated for every tree. Wild fruits were collected when mature (with a yellowish to brownish color). Fruits from homegardens were collected from the trees only if recognized as mature by the owners, but although the pericarp was lignified, seeds were not physiologically mature, which prevented us from including seed size (or seed weight) in our analysis.

We measured the following vegetative characters of trees: total height (in m), trunk height (from the ground to the main branching point, in cm), trunk perimeter (in cm, measured midway between the ground and the first main branch, as these trees ramify early), number of main branches departing from the trunk, crown area (in m2, calculated as an ellipsoid from two crown diameters N-S and E-W) and mean number of leaves per bundle (from a random sample of 3–5 bundles per tree; see Figure 3). For evaluating leaf shape a random sample of 10 leaves per tree was collected from one branch facing to north, and their shape characterized with Elliptic Fourier Descriptors analysis (EFDs). This method analyzes the contour shape as a sum of waves or harmonics, each of them defined by four EFDs, which can be subject to multivariate analyses to summarize the morphological variation of the sample [44-47]. After being scanned at 150 dpi with a 3x3 cm reference, leaves’ shape was characterized using the software Shape ver. 1.3 [48]. In order to define a good number of harmonics for our sample, we calculated the percentage of pixels that differed between the original contour and the digital contour based on 1 to 20 harmonics; we found that seven harmonics allowed for the reconstruction of 97% of the original contour (data not shown). We calculated the mean leaf shape for every tree and performed a centered non-scaled principal component analysis (PCA) to obtain two scores of leaf shape based on the first and second principal components. Differences in leaf shape between varieties were evaluated through ANOVA of PCA scores (in R version 2.15.1, available online from the R Foundation for Statistical Computing). Mean leaf area was also calculated for each tree. Vegetative characters were analyzed in a total of 136 trees (59 wild, 77 from homegardens).

In order to describe the characteristics of each variety, we calculated the mean and standard deviation as well as the coefficient of variation (CV) for fruit and vegetative characters; differences between varieties were evaluated with non-parametric Wilcoxon’s sum rank test with a Bonferroni correction for multiple comparisons.

In order to identify those characters with higher capacity of separating varieties, a linear discriminant analysis (LDA) was applied to fruit and vegetative characters based on five groups: *uas* wild variety growing in the flooded savanna (*uas*S), *uas* wild variety growing in homegarden (*uas*H), *luch* domesticated variety, *sac luch* domesticated variety and *yaax luch* domesticated variety (Table 1). The statistical significance of group separation was evaluated by Wilk’s lambda (λ). LDA was performed through IBM SPSS v.19.0.0®. All statistical tests considered a significance threshold of α = 0.05.

## Results and discussion

### Current uses of *Crescentia cujete* bowls in lowland Maya culture

**Figure 4:**
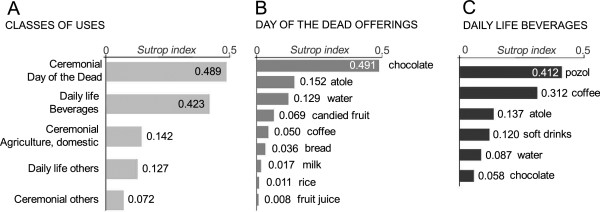
**Culturally relevant uses of *C. cujete *bowls in the study area. A)** Salience index of each of five classes of ceremonial and daily life uses of bowls. **B)** Salience index of food and beverages mentioned in the data subset on Day of the Dead offerings (total of lists belonging to this class: 30); **C)** Salience index of the subset of daily life beverages consumed in bowls (total of lists in the class: 26). For a description of the beverages and food items, see the main text.

Among the different classes of uses of bowls, ceremonial uses had a total frequency of 56 mentions, while uses of daily life were mentioned 35 times in total. Ceremonial offerings for the Day of the Dead and consumption of daily life beverages were the two classes with the highest salience index (*S* = 0.489 and 0.423, respectively), followed by agricultural and domestic offerings, other daily life uses and festivities (Figure 4A).

In the case of ceremonial offering of food and beverages during the Day of the Dead, chocolate was the item with the highest salience (*S* = 0.491) followed by *atole*, water and candied fruit (papaya *Carica papaya* L. or ciruela *Spondias purpurea* L.); other items such as coffee, bread, milk, rice and fruit juice were mentioned with less frequency or in lower ranks (Figure 4B). Interestingly, 20% of interviewees specified that they used bowls of different size according to the motive of the offering: small bowls are used when it is dedicated to children (November 1^st^) and big bowls when it is to adults that have passed away (November 2^nd^). Additionally, 42.5% of the interviewees said that for these offerings bowls must be new or exclusively used for this purpose (i.e. stored apart from others).

As for agricultural rites, 8 interviewees made explicit reference to the *ch’a chaak* ceremony (rain petition) where *saka’* and *balche’* are served in *jícara. Saka’* is a beverage made from maize cooked in plain water, sometimes sweetened with honey [37], while *balche’* is a fermented beverage made from the bark of *Lonchocarpus* spp. soaked in water with honey [49]. Additionnally, 8 interviewees mentioned the offering of *saka*’ during *hanli kol*, *wahi kol* or *primicia* (first-fruit ceremony), when burning the bush for establishing the *milpa* or for protecting it. Four interviewees mentioned that *saka’* is prepared in a big bowl and then distributed in smaller bowls. Offering of *saka’* in the domestic setting is oriented to appease the evil winds that cause illnesses in children and backyard animals. Other festivities mentioned by the interviewees were children school parades when *jícaras* are hit with one another for making a rhythmical sound, and a dance known as *Danza de la Cabeza de Cochino* (“Dance of the Pig’s Head”) by which the vow to a saint is renewed [50] and when *jícaras* are used as rattles.

Among daily beverages consumed in *jícara*, the most salient was *pozol* (*S* = 0.412), which is a mixture of water and dough made with maize cooked with lime (also known as *nixtamal*), mainly consumed by men during their work in the field. The second most salient beverage was coffee, followed by *atole* (corn-meal gruel), soft drinks, water and chocolate (Figure 4C).

**Figure 5:**
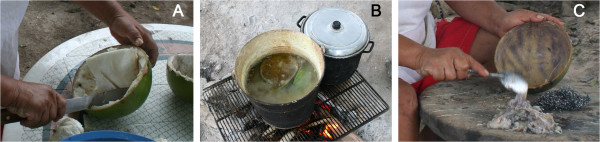
**Preparation technique of *jícaras *or bowls made from *C. cujete *fruits.** Fruits are collected when mature; at this stage, fruit’s colour turns from deep green to pale green or yellow-green; the fruit loses its shiny aspect and if pinched with a fingernail, no mark remains in its surface (this can be interpreted as the pericarp being already lignified; if the fruit is immature, bowls are too soft and get deformed when drying). After cutting the peduncle as short as possible, the two halves are defined in the fruit’s surface with the help of a knife’s point and a thread that surrounds the fruit vertically (always from the scar of the peduncle through the base of the fruit). The fruit is then cut with a fine-toothed saw following the line mark. Pulp is extracted and thrown away **(A)**. The halves are boiled in water with lime from 5 to 20 minutes, until the remaining pulp attached to the pericarp is soft (too much time in the water will propitiate an oxidation of the pulp, which would leave a black, undesirable stain in the bowl) **(B)**. The pulp is immediately scrapped off with a spoon or an easy-open can end **(C)**. Bowls are then washed with clean water and let drying upside-down for a couple of days outdoors.

When asked about the preparation technique for making bowls from *Crescentia cujete* fruits, 78% of men and 85% of women interviewed described a similar technique (Figure 5). Nine (9) interviewees agreed that both can equally prepare the bowls, although 11 interviewees specified differential gender tasks: men take the fruits from the tree and cut them in half, whereas women clean them.

A total of 17 interviewees (42.5%) said that they commercialize bowls, 4 of them specifying that selling was for the Day of the Dead offerings. Prices differed according to the informant, but in all cases they were associated to bowl size: from ¢50 to $3 Mexican pesos (USD ¢4 to ¢23) one small bowl, $1.5 to $5 (USD ¢12 to ¢38) a medium-sized one, and $5 to $10 (USD ¢38 to ¢77) the big ones. Fresh fruits may also be commercialized, but only 3 people interviewed (7.5%) said to have sold them to people who know how to prepare bowls ($3 Mexican pesos each, USD ¢23). Additionally, 4 interviewees (10%) said to have practiced *medianía*: they receive fresh fruits from someone owning a tree, they prepare the bowls and return to the owner one half of them, keeping the other half. One interviewee, who owns two trees, prepared 120 bowls before the Day of the Dead, working together with 3 members of his family during 4 days; if sold at a standard price, this activity may represent a total income of 28 to 46 USD (the equivalent of 6 to 10 days of work with the minimum salary). Individuals who practice *medianía*, those who have two or more trees in their homegardens, and those who commonly sell bowls at this date are generally known by the community members to be specialized in bowl’s preparation and commercialization.

Our results show that in the study area, *Crescentia cujete* bowls are used for a wide range of purposes linked to the life and culture of the Maya people from the Yucatan Peninsula, from everyday life uses to specialized practices associated to religious and agricultural rituals, despite the availability of plastic and glass containers. Daily consumption of *pozol* in jícara, in the same way as five centuries ago (recorded by bishop Diego de Landa cited in the Background section), shows the relevance of these bowls in the traditional way of life of these communities. At the same time, the presence of coffee and soft drinks supports the flexibility of such uses; it is also the case in ritual occasions, given that coffee and bread have been added to the list of foods consumed at contemporary Maya sacramental meals (Figure 4B; [51]).

The religious ritual of offering food and drinks to the souls of those who have passed away is undoubtedly one of the most important incentives for continuing the use of these natural bowls and, therefore, for the conservation of the species in homegardens, especially because there exists an economic dynamic linked to it. The idea of using bowls that are new or set apart, as it is the case for other objects of the offerings, expresses respect for “that which is holy, that which is effective in dealing with the gods” [52]. Chocolate is also strongly associated to their culture: archaeological and documentary evidence shows that the Maya have a long, continuous history of consuming liquid chocolate since the Preclassic period (600 BC, [53]) mainly in ceremonies of religious and political significance [51,54]. The consumption of *atole* was also common during feasting events [51].

Bowls occupy a central role as containers during agricultural ceremonies for the offering of *saka’* and *balche’*. These are beverages used exclusively for ritual purposes (for additional details see [51,52,55-58]) but, in contrast to the Day of the Dead offerings that occur in the domestic setting and involve the active participation of women, agricultural ceremonies occur in the *milpa*, which is a space commonly associated to men and located at the edge of the village or within nearby forest areas [55]. The belief in forest spirits (*aluxes, dueños del monte, señores del monte*) is widespread in these communities and the rituals observed to appease and thank them (such as *wahi kol* or *hanli kol*) are essential for assuring a good harvest [29]; rituals are also necessary to renew the divine permission for hunting [58].

The making and using of these natural containers is also closely linked to the maintenance of social relationships through daily and ritual situations among the Kari’na and Mayana people of Surinam, who use them for drinking *kasiri*, a fermented beverage made from cassava, and among the Maroons from that same region, who use *C. cujete* bowls for ritual bathing, preferring them over plastic cups [59].

Our findings show that there has been a continuous link between *jícaras* and traditional beverages from pre-Hispanic times to our days. In consequence, *C. cujete* has been managed and its fruits constantly selected by the Maya people for centuries. As long-lived perennials have long generation times and high outcrossing rates [60-63], they require a constant selection pressure that spans many human generations to achieve noticeable results. In the case of species whose fruits are edible, human selection has been mainly directed to obtain larger fruits with less toxic compounds and defensive structures [64], an expected pattern associated to their use as food, with emphasis on particular fruit characteristics depending on their final purpose. However, the role of tradition is outstanding in the case of *C. cujete* because its non-edible fruits cannot be expected to evolve in the same direction as edible species. In the case studied, it is the cultural relevance of its specific uses that has allowed for the constant selection pressure behind its domestication as we discuss in the section below, a pattern that seems to hold in other areas of the species distribution.

### Bowl uses and selection over the phenotypic variation of the species

**Figure 6:**
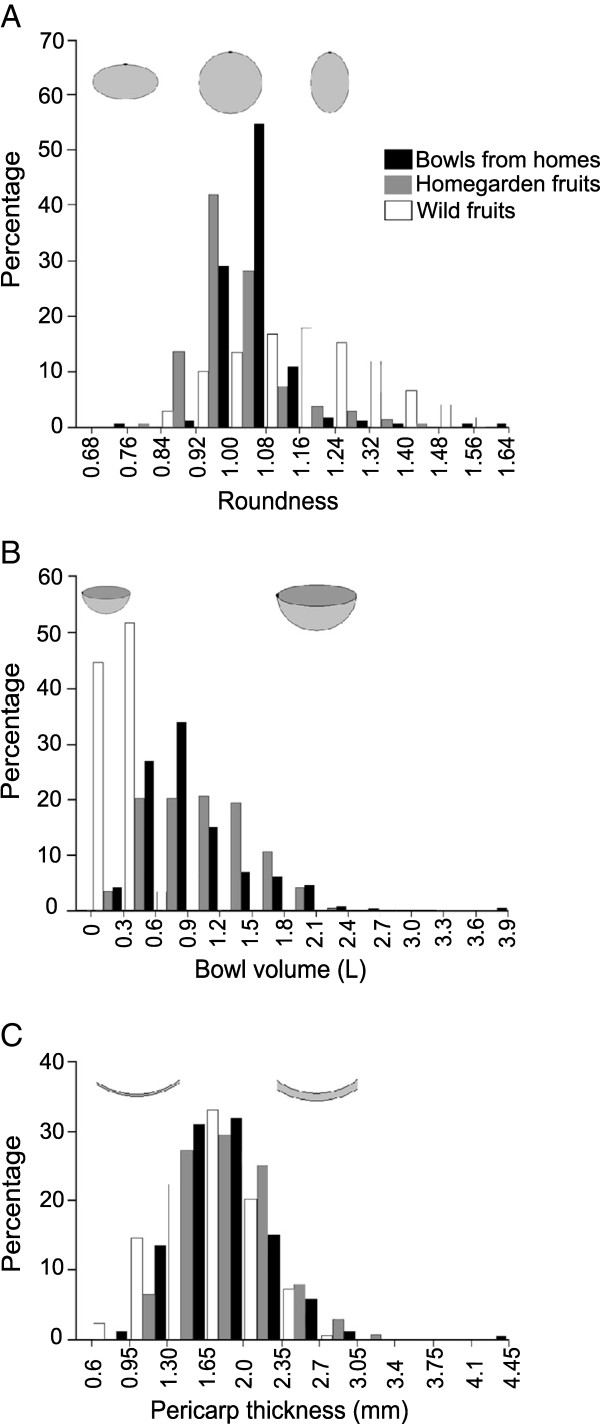
**Comparative distribution of characters among bowls in use from the houses visited, homegarden fruits and wild fruits (*n*** **= 258, 139 and 179, respectively). A)** Bowl’s roundness index; **B)** volume (for fruits, data from one half); and **C)** pericarp thickness. Drawings are not to scale, but included to show trends.

Bowls measured in homes had a mean roundness index close to a nearly-perfect half-sphere (mean ± sd *rd* = 1.033 ± 0.077); this was the less variable feature (CV = 7.4%) and the one with the higher degree of shift in relation not only to wild savanna fruits, which are more elongated (*rd* = 1.201 ± 0.15), but also in relation to homegarden fruits (*rd* = 1.007 ± 0.103; Figure 6A). Their volume distribution (*V* = 0.866 ± 0.497 L) is less shifted than roundness in relation to the available phenotypic variation of the species and presents a higher degree of variation (CV = 57.3%), but 61% of them are between 300 and 900 ml (Figure 6B). The thickness of their pericarp (*pt* = 1.73 mm ± 0.431) shows a smaller degree of variability than volume (CV = 24.9%), but its distribution is very similar to the one shown by homegarden fruits (Figure 6C).

The marked preference for round shapes in household bowls agrees with our initial hypothesis, but it is worth noticing that the shift among the three categories of samples (bowls from households, homegarden fruits and wild fruits) shows that two steps are involved in this process of human selection: the first one occurs when homegarden trees are preferred over wild trees and a second one when a subsample of homegarden fruits is processed into bowls. However, as *C. cujete* propagation is mainly based on tree cuttings and not on seeds from particular fruits (Table 1), it is the first step that has the major impact in artificial selection and the phenotypic differentiation of wild and domesticated populations. Our hypothesis also considered that selection of bowls would be focused on larger sizes, but their use for consuming beverages limits this trend, as consumption of any drink is commonly less than 1 L. Scarcity of large bowls in some homes could also be related to the fact that they have a higher price in the local market. Yet, it is important to notice that interviewees said that selling was mainly associated to the Day of the Dead offerings and, as we mentioned, the ritual use of the bowls favours a variety of sizes, including small and medium-sized bowls.

When we asked to people which variety the bowl belonged to, only 12 informants gave a specific answer; the rest of the sample was therefore assigned to the category “variety not determined”. From all the bowls surveyed, 12.8% were identified by our interviewees as *yaax luch* variety, 7.8% as *sac luch* variety, 2.7% as *luch* variety, 1.6% as *uas* variety, while 75.2% were from a non-determined variety. However, based on their shape and size, these non-identified bowls were most probably obtained from domesticated trees but not wild trees (Figure 6). Indeed, the *uas* wild trees from the savanna are not harvested while the *uas* wild trees are tolerated in homegardens and harvested although at low frequency (Table 1) and, given that they represent less than 30% of the trees found in the homegardens surveyed, they certainly provide a small proportion of the bowls used in the households.

### Morphological differentiation of local varieties

**Figure 7:**
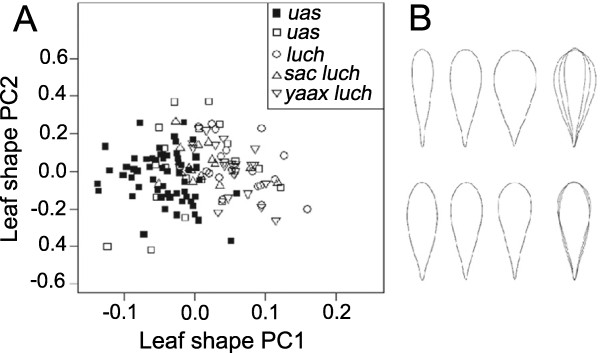
**Leaf shape variation in *C. cujete *varieties. A)** PCA of elliptic Fourier coefficients of leaves from trees growing in wild (black symbols) and homegarden (white symbols) sites. Each point represents the mean leaf shape of one tree, based on 10 leaves. **B)** Graphical representation of leaf shape variation through PC1 (top) and PC2 (bottom). The first three leaves represent (from left to right) mean leaf shape less one standard deviation, mean leaf shape, mean leaf shape plus one standard deviation; all three are superimposed in the rightmost figure.

Leaf shape variation identified by PCA of elliptic Fourier descriptors (Figure 7) shows that PC1 was associated to leaf’s width, as leaves from wild trees were oblanceolate while those from homegarden trees were obovate (see also Figure 1); PC2 reflected leaf’s base from cuneate to attenuate shapes. The ANOVA of the scores between varieties revealed significant differences in the first principal component (F_4,31_ = 31.35, *p* < 0.001) but not in the second one (F_4,31_ = 1.11, *p* = 0.35).

As described in Table 3, *uas* wild trees from savanna populations had small fruits, with elongated shapes, thin pericarp, long and narrow peduncles and abundant seeds. They were the largest among the varieties measured and their leaves were small, with narrow and elongated shape (Figure 7). Among the varieties found in homegardens, trees identified as *uas* (i.e. wild) produced fruits with significantly shorter and wider peduncles and fewer seeds than *uas* wild trees from the savanna, but their volume, shape and pericarp thickness was not significantly different from them (Table 3).

**Table 3:** Fruit characters of each variety from wild and homegarden trees

**Habitat**	**Savanna**	**Homegarden**			
**Variety**	***uas***	***uas***	***luch***	***yaax luch***	***sac luch***
	**(wild)**	**(wild)**	**(domesticated)**	**(domesticated)**	**(domesticated)**
**No. of trees**	60	11	9	16	18
**No. of fruits**	179	27	24	39	49
**Fruit volume (L)**	0.664 ± 0.207^a^	1.006 ± 0.469^a,A^	2.184 ± 0.733^B^	2.155 ± 0.840^B^	2.267 ± 0.795^B^
	31.2%	46.6%	33.6%	39.0%	35.1%
**Roundness**	1.201 ± 0.150^a^	1.132 ± 0.118^a,A^	0.979 ± 0.038^B^	1.000 ± 0.058^B^	0.960 ± 0.041^B^
	12.5%	10.4%	3.9%	5.8%	4.25%
**Pericarp thickness (mm)**	1.75 ± 0.38^a^	1.65 ± 0.28^a,A^	2.04 ± 0.48^A,B^	2.00 ± 0.22^B^	1.761 ± 0.292^A,B^
	21.8%	16.7%	23.3%	10.9%	16.6%
**Peduncle length (mm)**	43.10 ± 7.45^a^	29.11 ± 9.63^b,A^	16.45 ± 4.75^B^	15.38 ± 3.71^B^	15.796 ± 3.189^B^
	17.3%	33.1%	28.9%	24.1%	20.2%
**Peduncle width (mm)**	6.09 ± 0.96^a^	7.05 ± 0.76^b,A^	9.25 ± 1.40^B^	9.31 ± 1.15^B^	9.217 ± 1.008^B^
	15.8%	10.7%	15.2%	12.4%	10.9%
**Seed number**	571.2 ± 215.6^a^	342.1 ± 120.7^b,A^	398.6 ± 109.6^A^	480.5 ± 153.9^A^	448.78 ± 174.15^A^
	37.8%	35.3%	27.5%	32.0%	38.8%

Means ± sd are shown in each line above, and the CV below. ^a,b^: statistical difference for each character according to Wilcoxon’s sum rank test between *uas* from savanna and *uas* from homegarden; absence of significant difference is shown with the same lowercase letter. ^A,B^: statistical difference for each character among varieties growing in homegardens; absence of significant difference is shown with the same uppercase letter. For all tests alpha = 0.007 according to a Bonferroni correction.

As shown in Table 4, trees of domesticated varieties found in homegardens were generally shorter, thinner, with lower trunks, smaller crown areas and less leaves per bundle, although they were not significantly different from the wild variety growing in homegardens. Yet, they had lower total tree heights and smaller crown areas (W = 489.5, *p* < 0.001; W = 683, *p* < 0.001, respectively) than wild trees from the savanna. All domesticated varieties were characterized by a larger leaf area and a more rounded leaf shape, significantly different from *uas* wild trees growing in homegardens (Table 4; see Figure 1B); fruit morphology was not significantly different among them in any of the variables analyzed but when compared to the wild variety (*uas*) growing in homegardens, their fruits had significantly larger volumes, rounder shapes, shorter and wider peduncles (Table 3).

**Table 4:** Vegetative characters of each variety from wild and homegarden trees

**Habitat**	**Savanna**	**Homegarden**			
**Variety**	***uas***	***uas***	***luch***	***yaax luch***	***sac luch***
	**(wild)**	**(wild)**	**(domesticated)**	**(domesticated)**	**(domesticated)**
**No. of trees**	59	22	18	22	15
**Tree height (m)**	5.51 ± 1.49^a^	5.02 ± 2.37^a,A^	2.78 ± 0.96^B^	3.80 ± 1.19^A,B^	4.04 ± 1.24^A,B^
	27.0%	47.2%	34.5%	31.3%	30.7%
**Trunk height (m)**	0.59 ± 0.46^a^	0.89 ± 0.55^a,A^	0.62 ± 0.44^A^	0.45 ± 0.38^A^	0.46 ± 0.41^A^
	77.9%	61.8%	71.0%	84.4%	89.1%
**Trunk perimeter (m)**	1.07 ± 0.38^a^	0.73 ± 0.53^b,A^	0.49 ± 0.18^A^	0.63 ± 0.28^A^	0.57 ± 0.20^A^
	35.5%	72.6%	36.7%	44.4%	35.1%
**Crown area (m**^**2**^**)**	36.37 ± 19.71^a^	26.23 ± 24.98^a,A^	10.70 ± 8.12^A^	23.36 ± 11.91^A^	18.52 ± 11.24^A^
	54.2%	95.2%	75.9%	50.9%	60.7%
**No. main branches**	2.78 ± 0.93^a^	2.32 ± 0.57^a,A^	2.22 ± 0.65^A^	2.36 ± 0.66^A^	2.27 ± 0.46^A^
	33.5%	24.6%	29.3%	28.0%	20.3%
**No. leaves/bundle**	6.25 ± 1.60^a^	5.45 ± 2.19^a,A^	4.20 ± 1.39^A^	4.42 ± 1.97^A^	4.33 ± 2.15^A^
	25.6%	40.1%	33.1%	44.6%	49.6%
**Leaf area (cm**^**2**^**)**	9.38 ± 2.70^a^	18.04 ± 12.35^b,A^	24.85 ± 15.94^A,B^	31.09 ± 15.30^B^	30.05 ± 8.55^B^
	28.8%	68.5%	64.1%	49.2%	28.5%
**Leaf shape PC1**	−0.041 ± 0.041^a^	−0.001 ± 0.057^b,A^	0.063 ± 0.043^B^	0.052 ± 0.035^B^	0.012 ± 0.042^A^
**Leaf shape PC2**	−0.003 ± 0.013^a^	0.002 ± 0.021^a,A^	0.002 ± 0.014^A^	0.001 ± 0.013^A^	0.003 ± 0.009^A^

Means ± sd are shown in each line above, and the CV below. ^a,b^: statistical difference for each character according to Wilcoxon’s sum rank test between *uas* from savanna and *uas* from homegarden; absence of significant difference is shown with the same lowercase letter. ^A,B^: statistical difference for each character among varieties growing in homegardens; absence of significant difference is shown with the same uppercase letter. For all tests alpha = 0.007 according to a Bonferroni correction.

**Figure 8:**
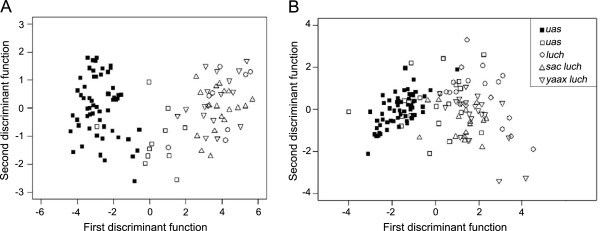
**Linear discriminant analysis between five categories of *C. cujete *trees growing in putative wild (black symbols) and homegarden (white symbols) sites, based on A) fruit characters (*n*** **= 114 trees) and B) vegetative characters (*n*** **= 136 trees).**

The first discriminant function of the LDA based on fruit characters accounted for 97.9% of the total variance and was significant (Wilk’s λ = 0.076, *p* < 0.001) showing a clear differentiation of wild and domesticated groups; the second discriminant function related to the discrimination among domesticated varieties accounted for an additional 1.4% of the variance and was not significant (Wilk’s λ = 0.818, *p* = 0.119). When considering vegetative characters, differentiation of groups by the first discriminant function accounted for 88.3% of the total variance and was statistically significant (Wilk’s λ = 0.210, *p* < 0.001); the second discriminant function was statistically significant but accounted for only 5.8% of the total variance (Wilk’s λ = 0.734, *p* < 0.05) (Figure 8).

According to the LDA, the most important fruit character for discriminating groups was peduncle length, followed by (in decreasing order) fruit volume, seed number, peduncle width, pericarp thickness and roundness. When considering vegetative characters, the most important variables were leaf shape (PC1 score), followed by leaf area, total height, trunk perimeter, leaf shape (PC2 score), number of main branches, number of leaves per bundle, trunk height and crown area. The weight of peduncle length in the LDA was unexpected, but there exists a strong correlation between larger fruits and shorter and wider peduncles, as these allow the fruit to remain attached to the branch despite being heavier (fruits with *V* = 4.3 L can weight 3200 grs; see Figure 1A) (Spearman’s correlation between weight and peduncle length ρ = −0.510, *p* < 0.001).

Our results show a pattern that can be observed for both fruit and vegetative characters: the first discriminant functions differentiate fundamentally wild trees from the savanna from trees growing in homegardens; domesticated varieties are not well differentiated from each other; and *uas* wild trees growing in homegardens occupy an intermediate position between *uas* wild trees from the savanna and the three domesticated varieties from homegardens (Figure 8).

As expected, we found a clear distinction of wild and domesticated varieties of *Crescentia cujete* based on fruit characters, but group separation based on vegetative characters was also statistically significant with leaf shape as the variable with highest weight for classifying such a distinction. Given that Maya local nomenclature is not based on leaf shape recognition and leaves should not be directly influenced by the selection on fruits, we consider this differentiation to be a secondary effect of the differential genetic identity of wild and domesticated varieties. Aguirre-Dugua et al. [13] documented that *uas* wild trees from savanna and homegardens harbour cpDNA haplotypes genetically distant from the cpDNA haplotype displayed by the domesticated varieties. This pattern of genetic identity suggests the presence of seed-mediated gene flow from wild populations into the homegardens and, in some cases, the pollination of domesticated trees by wild pollen. In consequence, we consider that the vegetative differences between trees growing in the savanna and in homegardens, and the intermediate position of *uas* wild trees tolerated in homegardens, reflect the genetic structure of the local populations of *Crescentia cujete*. Whether this association between leaf morphology and varieties is common in *C. cujete* is unclear. Arango-Ulloa et al. [65] report a diversity of leaf shapes in *C. cujete* trees from Colombia (lanceolate, oblong, spatulate, oblanceolate, obovate and elliptic), but they do not specify if they are associated to particular fruit morphologies (they report eight different fruit shapes) or local varieties.

Finally, we found domesticated varieties having smaller tree sizes (Table 4), this could be associated to their younger age (communities such as Chun Ek, Chanchén and Pachuitz being funded only five decades ago), to a different genetic composition, as previously mentioned, but it could also be due to management processes. Aguirre-Dugua et al. [13] documented that the majority of domesticated trees growing in homegardens were planted from clonal cuttings, and 6 people interviewed (15%) said that these must be planted horizontally, in such a way that the different branches develop from the ground, instead of having one trunk with a tendency of vertical growth. This practice would therefore favour a low-standing tree, which represents a selection trend common to other tree species under domestication [64] related to an easier harvest and independent of selection on fruit size.

### Differentiation of varieties and bowl uses

Given that our morphological analysis did not differentiate domesticated varieties among themselves, it is possible that fruit characters important for the Maya nomenclature of domesticated varieties were not well represented by our morphometric data, since external colour was not included in our analysis and the roundness index could not be the most appropriate. As long as qualitative variables are important for the local nomenclature of a species, the statistical analysis of morphological characters for differentiating local varieties of perennial plants remains a challenge [66-68]. Nevertheless, we previously reported [13] that both specific varieties (*sac luch* and *yaax luch*) are equally valued by the Maya people. We noted that most people interviewed did not identify clearly the varieties of bowls used in homes, which contrasts with the clear naming of the tree’s variety identity. This fact appears to reflect that the domesticated group is real whereas the differentiation of domesticated varieties is unclear after fruits have been processed into bowls. Moreover, the same variety can produce fruits of different sizes (Figure 1), which are then used in the different daily and ritual contexts previously described.

The weak association between varieties and particular uses could be related to two aspects: the unavailability of a larger morphological diversity of *C. cujete* in the area, which would represent a limiting factor external to the selection process, and the direction of human selection guided by cultural preferences, which is an internal factor. While only round fruits were identified in the homegarden trees from the study area, additional fruit morphologies of *C. cujete* are known from other regions in Mexico (e.g. elongated fruits in the states of Oaxaca and Chiapas used as spoons, pers. obs.), Colombia [65] and Surinam [59]. In this last country, Meulenberg [59] reported that the Maroon people distinguish up to 7 cultivars of this species, giving a name to each one that indicates its specific purpose (bowl, spoon, musical instrument, etc.), and indigenous people from the Kari’na tribe recognize and use as well 3 cultivars distinguished by their particular fruit morphology and use. When compared with other areas, the morphological diversity of *C. cujete* found in the lowland Maya area is limited. However, our study shows that the cultural preferences of the Maya favour round shapes from *luch*, *sac luch* and *yaax luch* varieties despite the availability of wild *uas* elongated fruits.

Introgression from wild relatives has been an important source of genetic enrichment and diversification of cultivated pools in perennial plants such as apple [69], avocado [70], grape [71] and olive [72], but for gene flow to be evolutionary relevant for domestication, the resulting seedlings have to be selected by humans. In our study case, evidence of gene flow between homegarden and wild trees include the presence of tolerated wild *uas* trees in the homegardens with intermediate vegetative and fruit phenotypes, and the presence of wild haplotypes in such individuals [13], but these trees are not favoured by the Maya. On the contrary, wild elongated fruits (which could be appreciated by the people from Surinam who have such a variety in their villages, for example) are consistently being discarded. Our results therefore suggest that the limited diversity of fruit morphology available in the study area is being reinforced by stabilizing selection toward rounder fruits, preventing the diversification of the cultivated pool and keeping all domesticated varieties as part of one single large group.

## Conclusions

Bowls made from *Crescentia cujete* fruits are used for a wide range of purposes linked to the daily and ritual life of the Maya people from the Yucatan Peninsula, and preferences associated to these uses are linked to human selection among one wild and three domesticated varieties of the species. *Jícaras* are mostly used as containers for traditional beverages such as chocolate and maize-based *pozol*, a long-standing association that dates back to pre-Hispanic times and that has allowed for the transgenerational human management of this long-lived tree species and the selection of its non-edible fruits. Men and women are both related to these containers, but in different spaces and ceremonies: men in agricultural and hunting rituals, women in domestic offerings and daily life. Bowl roundness is the most strongly selected character, whereas volume is important only in some cases, and such selection occurs in two stages: by favouring domesticated varieties with rounder and larger fruits in the homegardens, and then by choosing the best fruits from homegarden trees. Bowl uses were linked to fruit characteristics that define the differentiation of wild *versus* domesticated varieties. However, we did not find significant morphological differences among the three domesticated varieties nor a pattern of selection of different purpose cultivars; rather domesticated varieties form a large group where rounder bowls are selected from and where different fruit sizes meet different roles. This result is associated to a limited morphological diversity available at the local level but also to the strong inclination of the Maya for round fruits. Such preference exerts a stabilizing selection over the available phenotypic diversity of the species and prevents the diversification of the cultivated pool through introgression from wild trees, which produce elongated fruits, otherwise appreciated and used by other peoples in different areas of the species distribution. When compared to other domesticated long-lived perennial plants, domesticated *C. cujete* shows a pattern of larger fruit sizes and a moderate dwarfism that facilitates harvest.

As a whole, the domestication of *Crescentia cujete* and its cultural dimension are recently becoming explored, but the presence of this species among different peoples and geographical regions offers a promising possibility of developing comparative studies. Together with previous and current studies developed on the bottle-gourd *Lagenaria siceraria* (Molina) Standl. [73-75], the study of non-edible fruits will undoubtedly broaden our understanding of plant domestication through its natural and cultural components.

## Competing interests

The authors declare that they have no competing interests.

## Authors’ contributions

XA-D designed the research on *Crescentia cujete*, carried out field work, analysis and interpretation of data, and wrote the manuscript. EP-N conducted field work and data collecting. AC coordinator-supervisor of the main research project on plant domestication in Mesoamerica, participated in the conception of the study, and made substantial contributions to the manuscript. All authors read and approved the final manuscript.

## Authors’ information

XA-D PhD student of the Posgrado en Ciencias Biomédicas at the Centro de Investigaciones en Ecosistemas (CIEco), UNAM. EPN full time academic technician at CIEco. AC full time researcher at CIEco, UNAM.
